# A new flea beetle genus from sub-Saharan Africa, and taxonomic remarks on the *Blepharida* genera group in the Afrotropical region (Chrysomelidae, Galerucinae, Alticini)

**DOI:** 10.3897/zookeys.1228.139654

**Published:** 2025-02-20

**Authors:** Paola D'Alessandro, Maurizio Biondi

**Affiliations:** 1 Department of Health, Life and Environmental Sciences, University of L'Aquila, 67100 L'Aquila, Italy University of L'Aquila L'Aquila Italy

**Keywords:** *Afrotropicaltica*, *Blepharidina*, *Calotheca*, *Cladocerafulvipennis*, *Diamphidia*, new synonymy, *Polyclada*, *Xanthophysca*

## Abstract

*Cladocerafulvipennis* Jacoby is transferred to *Afrotropicaltica***gen. nov.**, here described. The new genus belongs to the group of Afrotropical genera traditionally attributed to the *Blepharida* group: *Blepharidina* Bechyné, *Calotheca* Heyden, *Diamphidia* Gerstaecker, *Polyclada* Chevrolat, and *Xanthophysca* Fairmaire. A diagnostic key to these genera, and the list of species currently attributed to *Diamphidia* and *Polyclada* are provided based on type material and original species descriptions. Species and genera whose taxonomic position needs a revision were identified. Finally, the following synonymy is proposed: *Cladocerafulvipennis* Jacoby, 1895 = *Blepharidafavareli* Achard, 1922, **syn. nov.**

## ﻿Introduction

The term *Blepharida* group refers to a group of approximately 21 genera from the Afrotropical, Nearctic, Neotropical, and Oriental regions (Furth 1992, 1998, Medvedev 1999; Furth and Lee 2000; Biondi et al. 2017; Calcetas and Staines 2024). Various authors discussed similarities and affinities between the genera based on adult and larval morphology, molecular markers, and ecology, and established new nomenclatural acts, generally focusing on one subset of taxa: Furth (1992, 1998), Medvedev (1999), Furth and Lee (2000), Becerra (2004), Takizawa (2005), Chaboo et al. (2007), Lee and Cheng (2007), Prathapan and Chaboo (2011), Biondi et al. (2017). Furth and Lee (2000) provided a morphological synthesis of the group based on adult characters (tarsal claws, procoxal cavities, head, pronotum, hind femora, eye, proepimeron, and metatibia) and larval characters (antenna, mandible, labrum, stemmata, endocarina, coronal suture, frontal suture), but highlighted that some characters are not shared by all the genera.

The Afrotropical region hosts five genera traditionally considered to belong to the *Blepharida* group, sensu Furth and Lee (2000): *Blepharidina* Bechyné, 1968; *Calotheca* Heyden, 1887; *Diamphidia* Gerstaecker, 1855; *Polyclada* Chevrolat, 1837, and *Xanthophysca* Fairmaire, 1901 (Biondi and D’Alessandro 2010, 2012; Biondi et al. 2017, 2022). Genus *Blepharidina* has recently been the object of a deep taxonomic revision (Biondi et al. 2017, 2019; D’Alessandro et al. 2018a, 2019). It includes 32 species, of which 12 are in the subgenus Afroblepharida Biondi & D’Alessandro, 2017, and 20 in the subgenus Blepharidina s. str. The genus mainly occurs in the intertropical area of Africa, with *Blepharidina* primarily distributed in the south and *Afroblepharida* largely in the central-eastern area, including Socotra Island, with extensions towards the north and in the west. Moreover, *Blepharidina* s. str. species generally occur in mesic environments, while Blepharidina (Afroblepharida) species are generally associated with more xeric conditions (D’Alessandro et al. 2018b; Iannella et al. 2021). *Calotheca* comprises 37 described species (Biondi et al. 2017; D’Alessandro et al. 2020, 2021, 2022, 2023a, 2023b). Some species groups have been revised recently, while others are still under investigation by the authors. The genus is widespread in sub-Saharan Africa, and particularly common in the eastern and southern parts of its distribution range, with limited extensions into Israel and the Arabian Peninsula (Iannella et al. 2021). *Calotheca* species are generally associated with savannas and forests, or with the typical South African vegetation, such as Albany thicket and Fynbos (D’Alessandro et al. 2018b; Iannella et al. 2021; Biondi et al. 2024). Many records reported *Searsia* species (Anacardiaceae) as the primary host plants (Koch 1958; Furth and Young 1988; MB, pers. data; E. Grobbelaar, pers. comm. 10 September 2024). *Polyclada* occurs in sub-Saharan Africa and the Arabian Peninsula with ~ 15 species (Biondi and D’Alessandro 2010, 2012; D’Alessandro and Biondi 2023). Species are associated with Anacardiaceae and Burseraceae and are found in various woodland and savannah ecosystems (Chaboo et al. 2007; Prathapan and Chaboo 2011; Biondi et al. 2022). *Diamphidia* comprises ~ 17 species, widespread in central, eastern, and southern-western Africa (Biondi and D’Alessandro 2010, 2012) and associated with *Commiphora* shrubs and trees (Burseraceae) (Chaboo et al. 2007; Biondi and D’Alessandro 2012). *Xanthophysca* is endemic to Madagascar, and currently includes 5–7 species (Biondi and D’Alessandro 2010, 2012; Biondi et al. 2017).

*Diamphidia*, *Polyclada*, and *Xanthophysca* have not been revised recently. *Polyclada* and *Diamphidia* species, previously described also under the generic names *Cladocera* Hope, 1840 and/or *Cladotelia* Kolbe, 1894, created a certain confusion in the generic name usage and, regarding *Polyclada*, also the genus author (Baly 1861; Chapuis 1875; Achard 1922; Laboissière 1941, 1942; Bryant 1942). In addition, discordances in the identification and description of key diagnostic characters for the genera *Diamphidia* and *Polyclada* have led to uncertainty in the attribution of some species and/or the transferring from one genus to the other (Baly 1861; Chapuis 1875; Achard 1922; Laboissière 1941, 1942; Bryant 1942).

In this paper, we establish the synonymy *Cladocerafulvipennis* Jacoby, 1895 = *Blepharidafavareli* Achard, 1922, syn. nov., designate the lectotype of *Blepharidafavareli*, and provide a reassessment of the taxonomic position of *Cladocerafulvipennis* by transferring it to *Afrotropicaltica* gen. nov. This new flea beetle genus has been established after the revision of the diagnostic characters of the Afrotropical genera traditionally attributed to the *Blepharida* group, specifically the adult morphological characters. Based on this revision, a diagnostic key to the genera is also provided. Since *Blepharidina* and most *Calotheca* species were revised and listed in recent papers (Biondi et al. 2017; D’Alessandro et al. 2018a, 2019, 2020, 2021, 2022, 2023a, b), particular attention is paid to *Diamphidia*, *Polyclada*, and *Xanthophysca*. The list of species attributed to these three genera is provided. Since the most recent taxonomic literature does not agree on some synonymies and/or the status of some taxa, possible synonyms are listed as separated species. Species whose generic attribution needs further investigation are reported. *Cladocera* and *Cladotelia* are currently considered as synonyms of *Polyclada* (Biondi and D’Alessandro 2012).

## Materials and methods

Material examined consisted of dried pinned specimens preserved in the depositories listed in the “Abbreviations” section; abbreviations followed the list on the website The Insect and Spider Collections of the World (Evenhuis 2021). Species are attributed to the genera *Diamphidia*, *Polyclada*, and *Xanthophysca* focusing on type material and original species descriptions. Species whose taxonomic position at the genus level needs a revision are listed separately. Exact label data are cited for all type specimens; a double slash (//) divided the data on different labels and a single slash (/) divided the data in different rows. Information included in square brackets has been added to the label data using the Google Earth website for coordinates and geographic information. Geographic coordinates for the localities were reported in Degrees and Decimal Minutes (DDM) format using the WGS84 datum. [?] refers to undetectable or doubtful locality or illegible handwriting. Specimens were examined, measured, and dissected using a Leica M205C stereomicroscope. Photographs were taken using a Leica DMC5400 camera and compiled using Zerene Stacker software, v. 1.04. Scanning electron micrographs were taken using a Hitachi TM-1000. Terminology for genitalia follows Döberl (1986), Schmitt and Uhl (2015: fig. 1), and D’Alessandro et al. (2016: figs 10E, 11E). Terminology for the metafemoral spring or metafemoral extensor tendon (Nadein and Betz 2016) follows Furth (1982).

### Abbreviations

#### Collections and depositories

**BAQ** Italy, University of L’Aquila, Collection of M. Biondi;

**MCZC**USA, Massachusetts, Cambridge, Harvard University, Museum of Comparative Zoology;

**MNHN**France, Paris, Muséum National d’Histoire Naturelle;

**MSNG**Italy, Genova, Museo Civico di Storia Naturale di Genova;

**NHMUK**United Kingdom, London, The Natural History Museum;

**NMPC**Czech Republic, Prague, National Museum (Natural History);

**RMCA**Belgium, Tervuren, Musée Royal de l’Afrique Centrale.

#### Biometrics

**LA** numerical sequence from base to apex of each antennomere, proportional to the length of the first antennomere;

**LAED** length of median lobe of the aedeagus;

**LAN** length of antennae;

**LB** total body length (from apical margin of head to apex of elytra);

**LE** length of elytra;

**LF** maximum length of hind femora;

**LP** medial length of pronotum;

**LSP** maximum length of spermatheca, including ductus;

**WE** maximum width of elytra combined;

**WF** maximum width of hind femora;

**WP** maximum width of pronotum.

### Taxonomic account

#### Afrotropicaltica

Taxon classificationAnimaliaColeopteraChrysomelidae

gen. nov.

0CDDD190-7E9A-5637-A96E-BECC248698A7

https://zoobank.org/011F6C24-9303-4535-9952-09533542B544

Fig. 1

##### Description.

**Figure 1.**
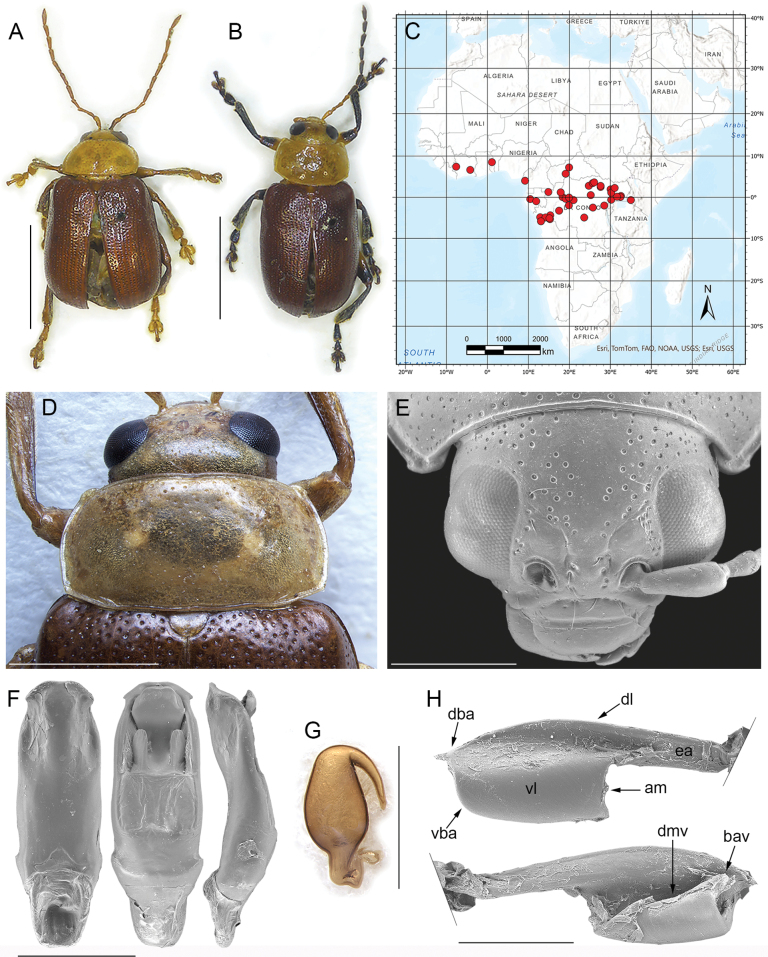
*Afrotropicalticafulvipennis* (Jacoby) comb. nov. **A** lectotype of *Blepharidafavareli* Achard, male, Ogooué, Sam-Kita, habitus **B** holotype of *Cladocerafulvipennis* Jacoby, male, habitus **C** distribution **D** head, pronotum, and base of elytra, male, Cameroun, Victoria **E** head, Republique du Congo, P.N. d’Odzala, Mbandza **F** median lobe of the aedeagus, from left to right in ventral, dorsal, and lateral view, Republique du Congo, P.N. d’Odzala, Mbandza **G** spermatheca, paralectotype of *Blepharidafavareli* Achard, Democratic Republic of the Congo, Eala **H** metafemoral extensor tendon, Camerun, Victoria. Abbreviations: am: apical margin; bav: basal angle of the ventral lobe; dba: dorsal-basal angle; dl: dorsal lobe; dmv: dorsal margin of the ventral lobe; ea: extended arm; vba: ventral-basal angle; vl: ventral lobe. Scale bars: 3 mm (**A**, **B**); 1 mm (**D**, **E**, **F**, **G**); 500 μm (**H**).

Body subelliptical-elongate in dorsal view, with subparallel sides (Fig. 1A, B), distinctly convex in lateral view. Dorsal surface glabrous, bicoloured in the only known species, with yellowish head and pronotum, and reddish brown elytra. Head (Fig. 1E) with frontal grooves, frontal carina, and frontal calli barely distinguishable; surface smooth or micropunctate, with rounded punctures approx. as large as the supraorbital setiferous pore; puncture absent on the middle of front and vertex; eyes large, ovoidal; minimum distance between eyes on vertex, as wide as 2 × the interantennal space; interantennal space prominent compared to the clypeus, as wide as ~ 2/3 the length of the first antennomere; maxillary palpi three-articulated, and labial palpi bi-articulated, both with slender, subcylindrical segments. Antennae (Fig. 1A, B) with 11 antennomeres, filiform, longer than 1/2 the body length in both sexes, but slightly shorter in female; antennomere II slightly shorter than 1/2 of antennomere I and as long as 1/2 of antennomere III; antennomere IV as long as (female) or slightly longer (male) than antennomere III; antennomeres V–IX generally slightly longer than antennomere I (male) or approximately as long as antennomere I (female); antennomere X slightly shorter than antennomere I, especially in female; antennomere XI distinctly longer than antennomere I. Prothorax distinctly depressed dorsally (not subcylindrical). Pronotum (Fig. 1D) subrectangular, slightly convergent anteriorly, with curved, moderately expanded lateral margins, clearly visible in dorsal view; basal margin moderately arched; surface smooth to micropunctate without any groove or depression; anterior angles barely prominent; posterior angles widely obtuse; punctation rounded, quite sparse and irregularly distributed. Scutellum subtriangular. Elytra (Fig. 1A, B, D) subparallel, with base as wide as pronotal base; lateral margins finely bordered, barely visible in dorsal view; punctation rounded, variably arranged in numerous rows or bands, or almost confused. Epipleurae oblique, clearly visible in lateral view. Procoxal cavities open posteriorly; prosternum approx. as wide as the procoxal cavities; intercoxal prosternal process quite narrow; mesosternum slightly wider than prosternum, and distinctly wider than mesocoxal cavities; metasternum wider than mesosternum, twice the width of the metacoxal cavities; first abdominal ventrite as wide as metacoxal cavities. Posterior femora elliptical-elongate, moderately swollen; dorsal margin of middle and hind tibiae with distinct ciliate emargination, acute apically; hind tibiae straight in dorsal view; apical spur of hind tibiae simple; third visible metatarsomere deeply incised; fourth visible tarsomere of metatarsus simple, not swollen in both male and female; claws appendiculate. Metafemoral extensor tendon (Fig. 1H) with extended arm (ea: Fig. 1H) as long as ~ 1/2 the dorsal lobe (dl: Fig. 1H); dorsal-basal angle (dba: Fig. 1H) approx. right angled, moderately prominent apically; ventral-basal angle (vba: Fig. 1H) distinctly obtuse and moderately rounded; apical margin of the tendon (am: Fig. 1H) slightly C-shaped; dorsal margin of ventral lobe (dmv: Fig. 1H) oblique; basal angle of ventral lobe (bav: Fig. 1H) quite close to the dorsal-basal angle of the tendon. Median lobe of the aedeagus (Fig. 1F) mostly subparallel in ventral view, with truncated apex, and curved in lateral view; dorsal ligula formed by a wider central lobe, and two lateral lobes. Spermatheca (Fig. 1G) with subovate basal part, narrowing towards ductus attach; distal part thin, without a distinct collum.

##### Type species.

*Cladocerafulvipennis* Jacoby, 1895.

##### Etymology.

The name of the new genus refers to flea beetle with strictly Afrotropical distribution. Gender feminine.

##### Distribution.

Cameroon, Central African Republic, Democratic Republic of the Congo, Gabon, Ivory Coast, Kenya, Republic of South Africa, Togo, Uganda (Fig. 1C).

#### Afrotropicaltica fulvipennis

Taxon classificationAnimaliaColeopteraChrysomelidae

(Jacoby, 1895), comb. nov.

6540888E-F736-5F22-8E82-1639B9EDD0D4

Fig. 1

Cladocera fulvipennis Jacoby, 1895: 179.
= Blepharidafavareli Achard, 1922: 8, syn. nov.
Blepharida fulvipennis (Jacoby, 1895): Achard 1922: 9, Bryant 1942: 161.
Diamphidia fulvipennis (Jacoby, 1895): Bechyné 1960: 111, Scherer 1969: 371.
Diamphidia fulvipennis favareli (Achard, 1922): Bechyné 1960: 111, Scherer 1962: 78, Scherer 1972: 16.
Diamphidia favareli (Achard, 1922): Laboissière 1941: 240, Biondi et al. 2017: 112.

##### Comments.

Achard (1922) described *Blepharidafavareli* and transferred *Cladocerafulvipennis* Jacoby to the genus *Blepharida*, due to the similarities with *B.favareli*. Bryant (1942) transferred *Cladocerafulvipennis* to *Blepharida*, as already proposed by Achard (1922). Bechyné (1960) established the combination *Diamphidiafulvipennis* (Jacoby, 1895) and considered the species described by Achard, *Blepharidafavareli*, as a geographic form of *D.fulvipennis*. Scherer (1969) erroneously reported *Diamphidiafulvipennis* (Jacoby, 1895) as *Diamphidiafulvipennis* Jacoby, 1893. We formalize here the synonymy between *Cladocerafulvipennis* Jacoby, 1895 and *Blepharidafavareli* Achard, 1922.

##### Type material examined.

***Holotype*** ♂ of *Cladocerafulvipennis*: “Type H.T // Togo / Africa // 42 // Type // Jacoby Coll. / 1909–28a. // *Cladocera* / *fulvipennis* / Jac. / Type” [8° 31.995'N 1° 5.853'E] (NHMUK). MCZC hosts the following specimen: “Type / 18327 // Boma / M. Tschoffen // Jacoby 2^nd^ / Coll. // fulvipennis Jac.” [Democratic Republic of the Congo, Boma, 5° 50.762'S 13° 4.296'E5°52'32"S, 13°02'00"E] (photos available at https://mczbase.mcz.harvard.edu/guid/MCZ:Ent:18527). Lectotype ♂ of *Blepharidafavareli* Achard, here designated: “Type // B. Favareli / Type / J. Achard det. // Ogooué / Sam-Kita” [Gabon, Samkita, 0° 26.516'S 10° 27.247'E] (NMPC). Paralectotype ♀ of *Blepharidafavareli* Achard, here designated: “Type // B. Favareli / Type / J. Achard det. // Fort-Sibut / Oubanghi-Chari” [Central African Republic, Fort-Sibut, 5°43.57140'N 019°05.11590'E] (NMPC). Paralectotype ♀ of *Blepharidafavareli* Achard, here designated: “Type // B. Favareli / Type / J. Achard det. // Musée du Congo / Eala / IX 1912 / R. Mayré” [Democratic Republic of Congo, Eala, 0° 2.441'N 18° 20.149'E] (NMPC).

##### Other material.

Cameroon • 16 specs, Victoria [= Limbé 4°1.917'N, 9°11.122'E], Jun.–Jul.1902, L. Fea leg. (MSNG). CENTRAL AFRICAN REPUBLIC • 1♀, Bamingui-Bangoran Pr., 45 km SSW Bamingui [7°16.400'N, 19°55.580'E], 450 m, 13–15 May 2009, A. Kudrna Jr leg. (BAQ). DEMOCRATIC REPUBLIC OF CONGO • 1 spec., Boma [5°50.762'S, 13°4.296'E], M. Tschoffen leg., Jacoby Coll. 1909-28a (NHMUK); 2 specs, ibid (RMCA); 1 spec., Eala [0°2.441'N, 18°20.149'E], Oct. 1935, J. Ghesquière leg. (RMCA); 1 spec., P.N. d’Odzala [1°16.167'N, 14°52.299'E], Mbandza, Nov. 1992, G. Carpaneto leg. (BAQ); 1 spec., Ekoiongouma, N1.20279 E17.88032 [1°12.16740' N, 17°52.81920' E], 341 m, 14 Feb. 2022, canopy light trap (-10 m), C.N. Hackfort & A. Tsoumou leg. (NHMUK); 1 spec., Lokandu [2°31.228'S, 25°44.566'E], Mar.1939, [Capt.] Marée leg. (NHMUK); 13 specs, ibid (RMCA); 1 spec., ibid, 1939 (RMCA); 3 specs, Equateur, Flandria [0°22.775'S, 19°5.500'E], 27 Oct.1932, R.P. Hulstaert leg. (RMCA); 1 spec., ibid, 31 Mar. 1932; 1 spec., Flandria, 15 Mar. 1932; 1 spec., ibid, 1931; 1 spec., Cataractes de Luozi [Luozi 4°54.711'S, 14°9.842'E], Dec. 1898, E. Luja leg. (RMCA); 1 spec., Bas-Congo, Lemfu [5°21.206'S, 15°15.709'E], Jun. 1945, Rév. P.L. De Reir leg. (RMCA); 1 spec., Tshuapa, Bokuma [0°40.648'S, 21°1.305'E], Dec. 1951, R.P. Lootens leg. (RMCA); 10 specs, Kisantu [5°7.473'S, 15°7.749'E], P. Goossens leg. (RMCA); 1 spec., ibid, ex col. Seeldrayers; 3 specs, ibid, 1905, col. Clavareau; 1 spec., Uelé, Dingila [3°38.633'N, 26°3.515'E], Jun. 1933, J.V. Leroy leg. (RMCA); 1 spec., Uelé, Paulis [= Isiro 2°46.212'N, 27°37.242'E], 1947, Abbeloos leg. (RMCA); 1 spec., Uelé, Bambesa [3°27.170'N, 25°41.579'E], 15 Sept. 1933, Lefèvre leg. (RMCA); 1 spec., Stanleyville [= Kisangani 0°30.542'N, 25°13.409'E], 1924, J. Ghesquière leg. (RMCA); 3 specs, Dima [3°16.47444'S, 17°28.99290'E], 14 Sept. 1908, A. Koller leg. (RMCA); 1 spec., Congo da Lemba [Lemba 4°24.598'S, 15°20.277'E], Jan. 1913, R. Mayné leg. (RMCA); 1 spec., ibid, 1–15 Apr. 1913; 7 specs, ibid, oct.–Dec.1911; 1 spec., Mongbwalu (Kilo) [1°55.640'N, 30°2.904'E], 1939, Mme Scheitz leg. (RMCA); 1 spec., Kilo [Kilo-Etat 1°49.738'N, 30°9.391'E], Dr. Abetti leg. (RMCA); 1 spec., Ganda Sundi [4°52.000'S, 12°52.000'E], [Comte J.] de Briey leg. (RMCA); 1 spec., Mahagi-Niarembe [between Mahagi and Niarembe 2°16.917'N, 31°3.371'E], 1935, Ch. Scops leg. (RMCA); 3 specs, Sud Ogowé, ‘Ngomo [4°57.378'S, 23°37.289'E], [H.A.] Junod leg. (RMCA); 2 specs, Bas Congo, Maydi [5°11.958'S, 15°9.195'E], 1945, Rév. P. Van Eyen leg. (RMCA); 21 specs, ibid; 28 specs, ibid, 1942; 2 specs, ibid, 1943; 3 specs, Kivu, Kavumu à Kabunga, km 82 (Mingazi) [2°1.870'S, 28°30.949'E], Apr.–Jul.1951, H. Bomans leg. (RMCA); 1 spec., ibid, Nov.–Dec. 1951; 1 spec., ibid, 1951; 1 spec., Bas-Uele, Buta [2°47.787'N, 24°44.473'E], 1926, Fr. Joseph leg. (RMCA); 1 spec., Mayumbe, Makaia N’Tete [Mayumbe 2°30.000'N, 27°37.000'E], 24 Nov. 1915, R. Mayné leg. (RMCA); 1 spec., Busira [0°5.233'S, 19°55.086'E], 18 Oct.1905, Waelbroeck leg. (RMCA); 1 spec., Equateur, Bohuma [1°8.099'N, 30°13.892'E], 1938, R.P. Hulstaert leg. (RMCA); 1 spec., Ilenge [1°58.940'S, 19°52.474'E], 30 Jan. 1913, R. Mayné leg. (RMCA). GABON • 1♀, Gabon Français [0°59.062'S, 11°54.238'E] (NMPC). IVORY COAST • 1 spec., Andé, Bongouanou [Bongouanou 6°39.047'N, 4°11.247W], Mar. 1962, J. Decelle leg. (RMCA); 1 spec., 1171 m, Mt Tonkoui Peak, 07°27'15.2"N, 07°38'12.5"W [7°27.230'N, 7°38.148'W], 1–8 Nov. 2015, general collecting, M. Aristophanous, P. Moretto, E. Ruzzier leg. (NHMUK). KENYA • 1 spec., Brit. E. Africa, Nyangori, N Kavirondo [0°41.195'S, 35°0.679'E], 4800 ft, 18–19 May 1911, S.A. Neave leg. (NHMUK). UGANDA • 1 spec., Mawakota [Mawokota region 0°36.800'S, 30°12.695'E], Nov. 1931, [V.G.L.] van Someren leg. (NHMUK); 1 spec., Boy [Bay (?)], Entebbe [0°3.290'N, 32°28.216'E], 2800-4000 ft, forest within four miles of Kitabi Hill, May–Jun. 1913, C.A. Wiggins leg. (NHMUK); 1 spec., Entebbe, 14 Aug. 1911, C.C. Gowdey leg. (NHMUK); 1 spec., Prot. Entebbe, 12–20 Jan. 1912, S.A. Neave leg. (RMCA); 1 spec., Kampala [0°19.253'N, 32°35.055'E], 1–10 Jan. 1918, C.C. Gowdey leg. (NHMUK); 1 spec., Shores of L. Isolt, or Wamala [Wamala 0°7.799'N, 31°38.115'E], 3800 ft, 7–8 Jan. 1912, S.A. Neave leg. (NHMUK).

##### Redescription.

Body subelliptical-elongate in dorsal view with parallel sides (Fig. 1A, B), distinctly convex in lateral view; total length of the body (LB) = 8.10 ± 0.91 mm (6.50 ≤ LB ≤ 9.70 mm) in male, and 8.77 ± 0.65 mm (7.52 ≤ LB ≤ 9.51 mm) in female; maximum pronotal width in the middle: WP = 3.43 ± 0.29 mm (2.86 ≤ WP ≤ 3.78 mm) in male, and 3.71 ± 0.25 mm (3.30 ≤ WP ≤ 3.98 mm) in female; maximum width of elytra in the middle: WE = 4.37 ± 0.37 mm (3.69 ≤ WE ≤ 4.85 mm) in male, and WE = 4.94 ± 0.42 mm (4.46 ≤ WE ≤ 5.72 mm) in female; WE/WP = 1.27 ± 0.04 (1.22 ≤ WE/WP ≤ 1.33) in male, and WE/WP = 1.33 ± 0.05 (1.29 ≤ WE/WP ≤ 1.44) in female.

Head and pronotum yellowish (Fig. 1A, B, D); elytra reddish brown; scutellum paler than elytra, often of the same colour as the pronotum or slightly darker; underside yellowish to pale brown; mouthparts yellowish, with black distal part of mandibles; antennae and legs with two colour patterns: both antennae and legs rather homogenous, yellowish to reddish brown, at most with slightly paler tarsi; antennal segments 1–4 yellowish and 5–11 blackish, and legs mostly blackish with the exception of femora, paler in their proximal part. Head (Fig. 1E) with smooth to micropunctate surface and sparse, rounded punctures, denser on vertex and near the inner ocular margins; frontal grooves and frontal calli barely distinguishable; frontal carina wide, not raised; eyes large, ovoidal; interantennal space as wide as ~ 2/3 the length of the first antennomere. Antennae (Fig. 1A, B) filiform, longer than 1/2 the body length: LAN = 6.07 ± 0.44 mm (5.43 ≤ LAN ≤ 6.69 mm) in male, and 5.62 ± 0.39 mm (5.04 ≤ LAN ≤ 6.31 mm) in female, and LAN/LB = 0.75 ± 0.05 (0.65 ≤ LAN/LB ≤ 0.84) in male, and 0.64 ± 0.03 (0.60 ≤ LAN/LB ≤ 0.68) in female. LA = 100:42:83:92:117:117:117:108:100:92:125 in male, and 100:42:83:83:108:100:100:92:83:83:117 in female. Pronotum (Fig. 1D) distinctly transverse: LP = 1.79 ± 0.17 mm (1.50 ≤ LP ≤ 1.94 mm) in male, and 1.85 ± 0.12 mm (1.65 ≤ LP ≤ 2.04 mm) in female, and WP/LP = 1.91 ± 0.06 (1.80 ≤ WP/LP ≤ 2.00) in male, and 2.01 ± 0.03 (1.95 ≤ WP/LP ≤ 2.05) in female; lateral margins slightly convergent anteriorly, moderately to distinctly curved, moderately expanded, visible in dorsal view; basal margin moderately arched; surface smooth to micropunctate; main punctation formed by rounded punctures, clearly impressed, quite sparse and irregularly distributed. Scutellum subtriangular, laterally rounded. Elytra (Fig. 1A, B, D) subparallel, clearly longer than wide, jointly rounded apically. LE = 6.57 ± 0.54 mm (5.43 ≤ LE ≤ 7.23 mm) in male, and 7.21 ± 0.48 mm (6.40 ≤ LE ≤ 7.86 mm) in female; WE/LE = 0.67 ± 0.02 (0.63 ≤ WE/LE ≤ 0.70) in male, and 0.68 ± 0.02 (0.66 ≤ WE/LE ≤ 0.73) in female; LE/LP = 3.66 ± 0.12 (3.50 ≤ LE/LP ≤ 3.91) in male, and 3.90 ± 0.09 (3.80 ≤ LE/LP ≤ 4.05) in female. Lateral margins finely bordered, indistinctly visible in dorsal view; surface very finely micropunctate or microwrinkled; main punctation clearly impressed, dense, variably arranged in numerous rows or bands, or almost confused. Humeral calli distinctly raised. Macropterous. Posterior femora moderately swollen (WF/LF = 0.44 ± 0.01); apical spur of hind tibiae very short; first tarsomere of fore- and middle legs clearly enlarged in male. Median lobe of the aedeagus (Fig. 1F) in ventral view: outline mostly subparallel with slightly sinuate margins, and narrower subapical part; apex truncated, distinctly expanded laterally; surface smooth, concave laterally and prominent medially in the apical third; in lateral view median lobe distinctly curved, with slightly sinuate outline; dorsal ligula formed by a wide central lobe, and two shorter and thinner lateral lobes, with base at apical ~ 1/3; LAED = 2.26 ± 0.10 mm (2.13 ≤ LAED ≤ 2.43 mm); LE/LAED = 2.90 ± 0.19 (2.55 ≤ LE/LAED ≤ 3.17). Basal part of the spermatheca (Fig. 1G) subovate, abruptly narrowing towards the ductus insertion; ductus short, thick, uncoiled, ventrally inserted; distal part of the spermatheca clearly bent towards the basal part, thin, acute apically; LSP = 0.93 ± 0.05 mm (0.87 ≤ LSP ≤ 1.02 mm); LE/LSP = 7.78 ± 0.25 (7.33 ≤ LE/LSP ≤ 8.11).

##### Distribution.

Cameroon, Central African Republic, Democratic Republic of the Congo, Gabon, Ivory Coast, Kenya, Republic of South Africa, Togo, Uganda (Fig. 1C).

##### Ecological notes.

Ecology, including host plant, unknown.

### Key to the Afrotropical genera of the *Blepharida* group

The combination of characters identifying *Blepharidina* and *Calotheca* was based on recent revisions (Biondi et al. 2017, 2019; D’Alessandro et al. 2018a, 2019, 2020, 2021, 2022, 2023a, 2023b). Regarding *Diamphidia*, *Polyclada*, and *Xanthophysca*, characters reported in the key were derived from the species whose genus attribution was unequivocal, due to their similarities with the type species.

**Table:** 

1	Antennae filiform, at most with distal segments slightly enlarged (Figs 1A, B, 2A, 3A, 6A); segment 4 approx. as long as 3	**2**
–	Antennae pectinate, serrate, or with middle segments clearly subtriangular (Figs 4A, 5A); segment 4 distinctly longer than 3	**5**
2	Pronotal punctation not homogenous, with at least some larger and more deeply impressed punctures arranged in lines (at least one line) or dense groups (Figs 2B, C, 3B, C)	**3**
–	Pronotal punctation uniform in size, homogenously or slightly irregularly distributed (Figs 1D, 4B, 5B, 6B)	**4**
3	Pronotum with main punctation arranged in two striae that are straight, L-, or C- shaped, running from the anterior margin towards the disc (ps: Fig. 3B, C); basal longitudinal furrows or small dimples are present in some species (bf: Fig. 3B). Frontal grooves elongate, sinuate, and deeply impressed from the superior ocular margin to the interantennal space (fg: Fig. 3E); frons surface not depressed between antennal sockets and clypeus. Extended arm of the metafemoral extensor tendon ~ 1/3 the length of the dorsal lobe; apical margin of the tendon clearly C-shaped (Fig. 3D)	***Calotheca* Heyden**
–	Pronotum with main punctation arranged in patches, and oblique, transverse, or longitudinal lines (Fig. 2B, C). Frontal grooves, not distinguishable (Fig. 2E), or at most short and moderately impressed; frons surface depressed between antennal sockets and clypeus. Extended arm of the metafemoral extensor tendon slightly shorter than 1/2 the length of the dorsal lobe; apical margin of the tendon moderately C-shaped (Fig. 2D)	***Blepharidina* Bechyné**
4	Procoxal cavities closed posteriorly. Pronotal punctation mostly uniform and dense (Fig. 6B). Frontal grooves distinctly impressed, from the antennal sockets to the upper ocular margin (Fig. 6B). Hind femur with a distinct emargination on the posterior side in both sexes, but more evident and tooth-shaped in male (Fig. 6A). Ductus spermathecae sub-truncate, practically absent (Fig. 6E). General shape of the median lobe of the aedeagus roughly subcylindrical (Fig. 6D). Metafemoral extensor tendon as in Fig. 6C	***Xanthophysca* Fairmaire**
–	Procoxal cavities open posteriorly. Pronotal punctation mostly uniform and sparse (Fig. 1D). Frontal grooves, if distinguishable, short and moderately impressed (Fig. 1E). Hind femur without a distinct emargination on the posterior side. Ductus spermathecae present (Fig. 1G). General shape of the median lobe of the aedeagus clearly flatter (Fig. 1F). Metafemoral extensor tendon as in Fig. 1H	***Afrotropicaltica* gen. nov.**
5	Eye large and generally distinctly convex (Fig. 5B). Antennae pectinate (generally in males) (Fig. 5A) or clearly serrate. Body distinctly elongate (Fig. 5A), with elytra subparallel laterally, or wider in the apical third. Extended arm of the metafemoral extensor tendon ~ 1/2 the length of the dorsal lobe; apical margin C-shaped (Fig. 5D). Median lobe of the aedeagus (Fig. 5C) straight in lateral view, with apical part thickset and bent ventrally. Spermatheca (Fig. 5E) with basal part roughly subcylindrical, and ductus apically inserted	***Polyclada* Chevrolat**
–	Eye small and moderately convex (Fig. 4B). Antennae with subtriangular or moderately serrate middle segments (Fig. 4A). Body generally robust, elliptical, more or less elongate, with elytra rounded laterally (Fig. 4A). Extended arm of the metafemoral extensor tendon distinctly longer than 1/2 the length of the dorsal lobe; apical margin widely arched (am: Fig. 4E). Median lobe of the aedeagus (Fig. 4C) slightly curved in lateral view, with apical part flat, depressed on the ventral surface, and softly curved ventrally. Spermatheca (Fig. 4D) with basal part generally slightly sinuate, dorsally bent at the ductus attachment	***Diamphidia* Gerstaecker**

**Figure 2.**
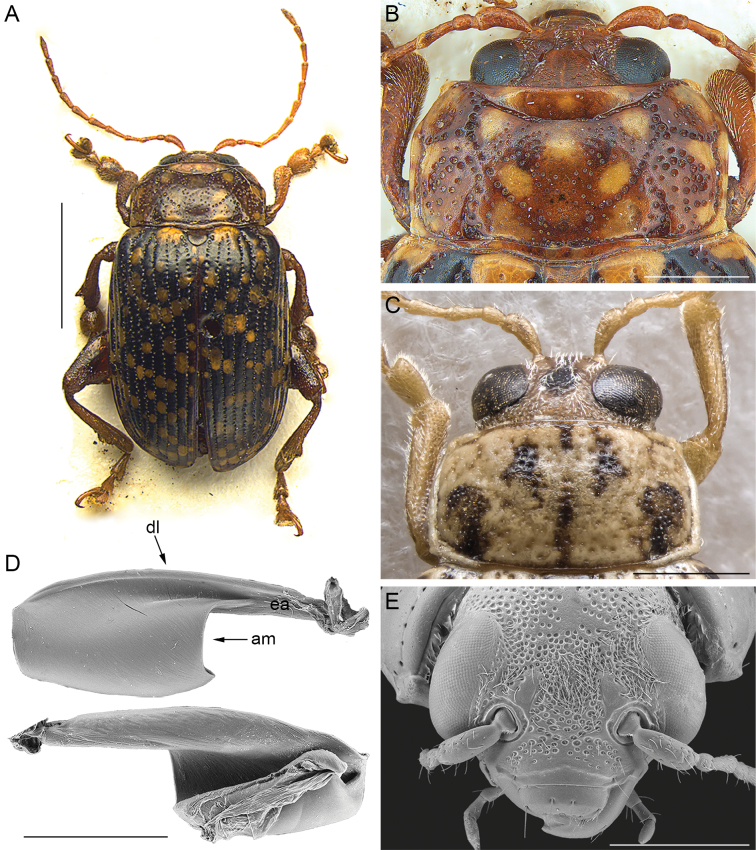
*Blepharidina* Bechyné **A** habitus of Blepharidina (Blepharidina) guttulata (Baly), male, modified from D’Alessandro et al. (2019)**B** ibid, head, and pronotum **C** head and pronotum of Blepharidina (Afroblepharida) gedyei, male, Kenya, Sosoma (BAQ) **D** metafemoral extensor tendon, modified from Biondi et al. (2017)**E** head of *Blepharidinaintermedia*, modified from Biondi et al. (2017). Abbreviations: am: apical margin; dl: dorsal lobe; ea: extended arm. Scale bars: 3 mm (**A**); 1 mm (**B**, **C**, **E**); 500 μm (**D**).

**Figure 3.**
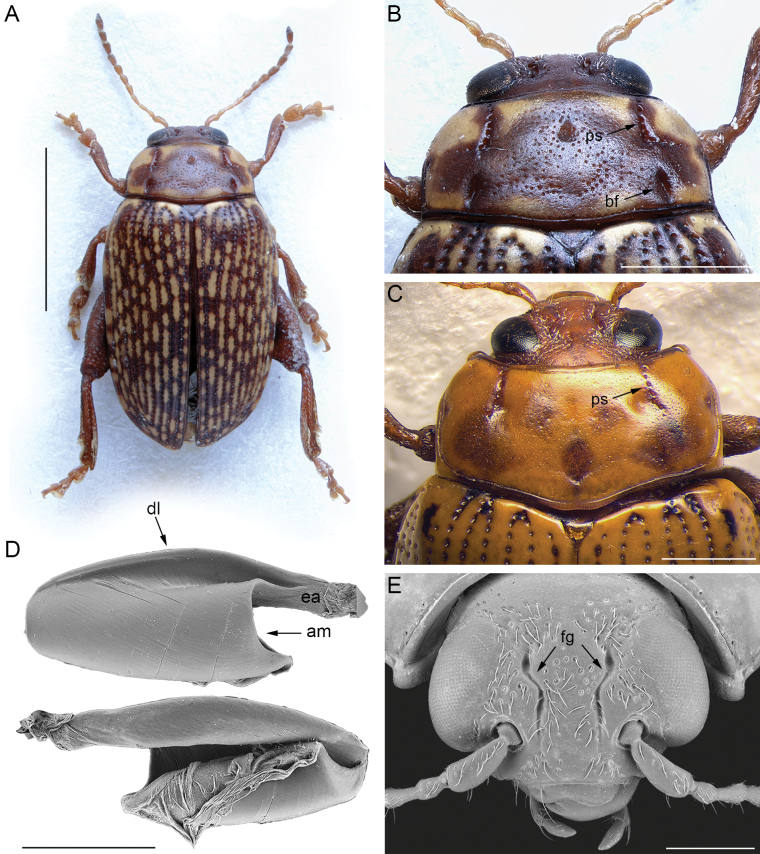
*Calotheca* Heyden **A** habitus of *Calothecaharoldi* (Baly), male, Republic of South Africa, Kranskloof (SANC) **B** ibid, head and pronotum **C** head and pronotum of *Calothecamarginalis*, modified from Biondi et al. (2017)**D** ibid, metafemoral extensor tendon **E** ibid, head. Abbreviations: am: apical margin; bf: basal furrow; dl: dorsal lobe; ea: extended arm; fg: frontal groove; ps: punctate stria. Scale bars: 3 mm (**A**); 1 mm (**B**, **C**); 500 μm (**D**, **E**).

**Figure 4.**
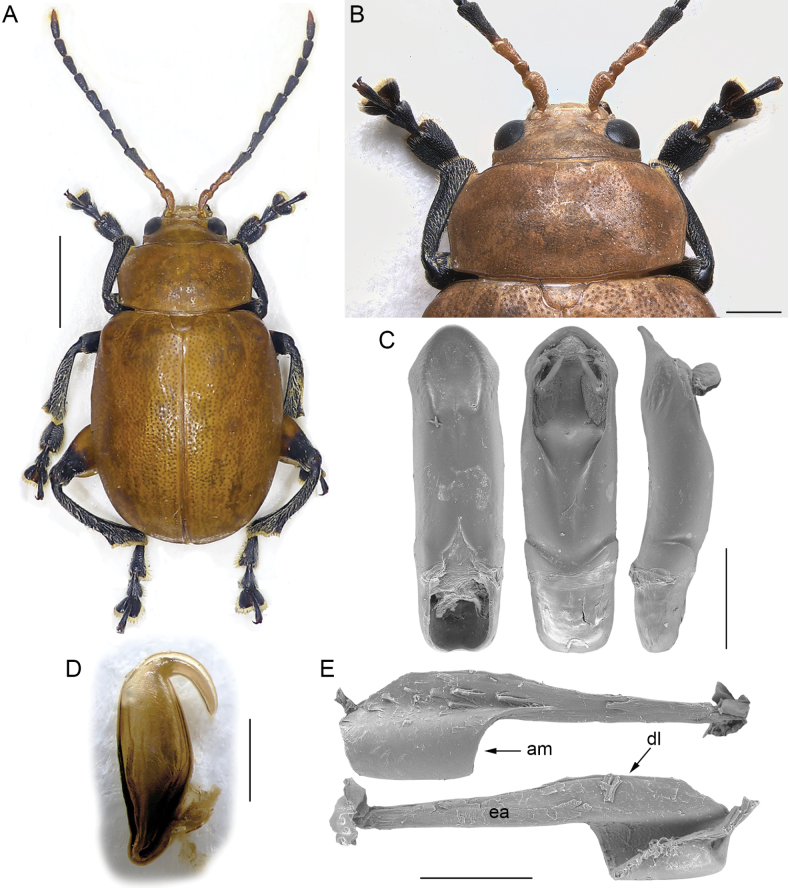
*Diamphidia* Gerstaecker **A** habitus of *Diamphidiafemoralis*, male, Mozambique, 25 km N Tete (NMPC) **B** ibid, pronotum and base of elytra **C** ibid, median lobe of the aedeagus, from left to right in ventral, dorsal, and lateral view, Zambia, Victoria Falls (BAQ) **D** ibid, spermatheca, Transvaal, Pretoria (MSNG) **E** ibid, metafemoral extensor tendon, Republic of South Africa, Blyde River Canyon (BAQ). Abbreviations: am: apical margin; dl: dorsal lobe; ea: extended arm. Scale bars: 3 mm (**A**); 1 mm (**B**, **C**); 500 μm (**D**, **E**).

**Figure 5.**
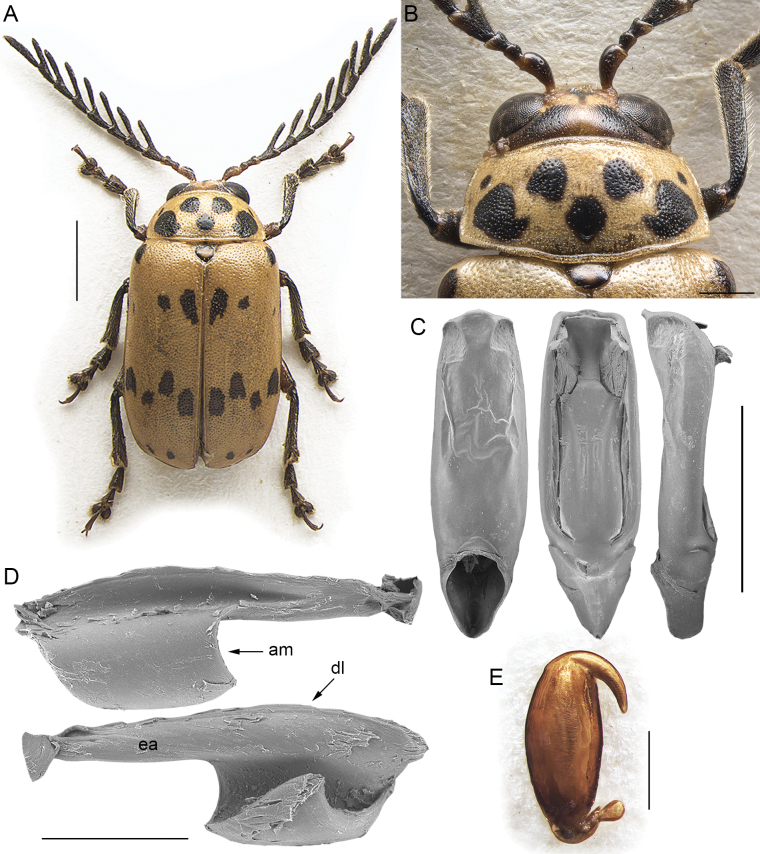
*Polyclada* Chevrolat **A** habitus of *Polycladapectinicornis*, male, modified from Biondi et al. (2022)**B** ibid, pronotum and base of elytra, male, Somalia, Bardera (BAQ) **C** ibid, median lobe of the aedeagus, from left to right in ventral, dorsal, and lateral view, modified from Biondi et al. (2022)**D** ibid, spermatheca **E** ibid, metafemoral extensor tendon, Tanzania, Mto. Wa Mbu (BAQ). Abbreviations: am: apical margin; dl: dorsal lobe; ea: extended arm. Scale bars: 3 mm (**A**); 1 mm (**B**, **C**); 500 μm (**D**, **E**).

**Figure 6.**
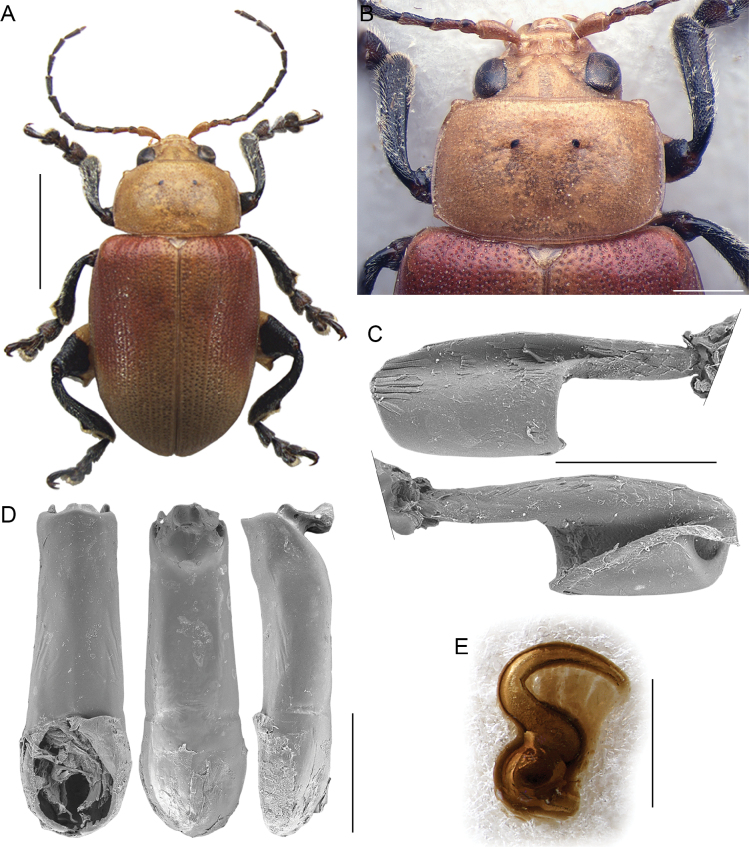
*Xanthophysca* Fairmaire **A** habitus of *Xanthophyscaperrieri*, male, modified from Biondi and D’Alessandro (2012)**B** ibid, pronotum and base of elytra **C** metafemoral extensor tendon, Madagascar Ouest, Antsingy (MNHN) **D** ibid, median lobe of the aedeagus, from left to right in ventral, dorsal, and lateral view **E** ibid, spermatheca, Madagascar, Ankarafantsika Rés. nat. (MNHN). Scale bars: 3 mm (**A**); 1 mm (**B**, **D**); 500 μm (**C**, **E**).

### List of *Diamphidia* species

*Diamphidiafemoralis* Gerstaecker, 1855, type species

*Diamphidiaangolensis* Jacoby, 1882

*Diamphidiabrevis* Laboissière, 1942

*Diamphidiaconcinna* Weise, 1906

*Diamphidiacurtula* Achard, 1922

*Diamphidiaflaveola* Laboissière, 1941

*Diamphidiaflaviceps* (Allard, 1887)

*Diamphidiajacobyi* (Gestro, 1895)

*Diamphidialesnei* Achard, 1922

*Diamphidialimbata* (Allard, 1887)

*Diamphidialocusta* Fairmaire, 1893

*Diamphidialongula* Weise, 1902

*Diamphidianigrifrons* (Allard, 1887)

*Diamphidianigripennis* (Jacoby, 1888)

*Diamphidianigroornata* Stål, 1858

*Diamphidiapatricki* Bryant, 1948

*Diamphidiarhabdoscelis* Laboissière, 1942

*Diamphidiarobusta* (Allard, 1887)

*Diamphidiarugipennis* Laboissière, 1942

*Diamphidiasemiopaca* Achard, 1922

*Diamphidiasimplex* (Peringuey, 1892)

*Diamphidiauniformis* (Jacoby, 1903)

*Diamphidiavittatipennis* Baly, 1865

### List of *Polyclada* species

*Polycladapectinicornis* (Olivier, 1791), type species

*Polycladabenti* Gahan, 1895

*Polycladabohemani* (Baly, 1861)

*Polycladacoriacea* Achard, 1922

*Polycladakenyensis* Bryant, 1942

*Polycladamaculicollis* Bryant, 1942

*Polycladaornata* (Baly, 1861)

*Polycladaornatipennis* Bryant, 1942

*Polycladasomaliensis* Bryant, 1948

*Polycladavariegata* (Weise, 1900)

### List of *Xanthophysca* species

*Xanthophyscaperrieri* Fairmaire, 1901, type species

*Xanthophyscaandroyana* Achard, 1915

*Xanthophyscadonckieri* Achard, 1915

*Xanthophyscakolbei* Weise, 1910

*Xanthophyscavariegata* Achard, 1915

The taxonomic position at genus level of some species is unclear because they lack one or more characters typical of *Diamphidia*, *Polyclada*, or *Xanthophysca*. These are *Polycladabedeli* Achard, 1922 with antennae of the *Diamphidia* type; *Polycladaflavipennis* Bryant, 1942 and *P.marginata* Bryant, 1948 with antennae of the *Diamphidia* type and aedeagus lacking the typical *Polyclada* characters; *Polycladaflexuosa* (Baly, 1865) with aedeagus different from the typical *Polyclada* species; *Diamphidiaornaticollis* Bryant, 1948, with closed procoxal cavities and filiform antennae.

## Discussion

*Afrotropicalticafulvipennis* (Jacoby) comb. nov. was referred to different genera in the previous publications (*Cladocera*, *Blepharida*, *Diamphidia*). However, based on its unique combination of characters, it is not attributable to any of the known flea beetle genera. Considering the diagnostic characters used to identify the main groups of Afrotropical flea beetles (Biondi and D’Alessandro 2012), *Afrotropicaltica* gen. nov. can be grouped with *Diamphidia*, *Blepharidina*, *Calotheca*, *Polyclada*, and *Xanthophysca*, traditionally attributed to the *Blepharida* group sensu Furth and Lee (2000). The following traits characterise these genera: antennae with 11 antennomeres; apical tarsomere of metatarsus simple (not swollen); dorsal margin of middle and hind tibiae with distinct ciliate emargination, which is acute or subrounded apically; prothorax distinctly depressed dorsally (not subcylindrical); first metatarsomere as long as the second or longer, wide, subtriangular or subrounded; body length generally ≥ 4.00 mm; claws generally appendiculate; pronotal sculpture variable but without any ante-basal transverse sulcus. Differences in antennae, pronotum, and elytra are helpful for genera and subgenera identification, as reported in the diagnostic key above.

Genus *Xanthophysca* lacks a comprehensive revision, but the diagnostic characters are coherent among the species attributed to it. Differently, some species currently attributed to *Polyclada* and *Diamphidia* show a combination of characters that makes them not referrable as *Polyclada* or *Diamphidia*. Therefore, *Xanthophysca* and especially *Diamphidia* and *Polyclada* need a revision, also based on the undetermined material and undescribed species present in public and private collections.

Furth and Lee (2000), while providing their morphological synthesis of the *Blepharida* group of genera, stated that some characters are not shared by all the taxa. This is true also for the genera discussed here. Assessing the monophyly of the group and the evolutionary affinity among the included taxa requires a more extensive investigation, also requiring molecular data. The same problems are present for assessing the existence of a wider *Blepharida* group clade.

## Supplementary Material

XML Treatment for Afrotropicaltica

XML Treatment for Afrotropicaltica fulvipennis

## Additional information

### Author ORCIDs

Paola D’Alessandro https://orcid.org/0000-0002-4481-9152

Maurizio Biondi https://orcid.org/0000-0003-2190-7376

### Data availability

All of the data that support the findings of this study are available in the main text.
